# Multilocation and Multiscale Learning Framework with Skip Connection for Fault Diagnosis of Bearing under Complex Working Conditions

**DOI:** 10.3390/s21093226

**Published:** 2021-05-06

**Authors:** Hongwei Ban, Dazhi Wang, Sihan Wang, Ziming Liu

**Affiliations:** School of Information Science and Engineering, Northeastern University, Shenyang 110819, China; 1900703@stu.neu.edu.cn (H.B.); 2010257@stu.neu.edu.cn (S.W.); 1900721@stu.neu.edu.cn (Z.L.)

**Keywords:** deep learning, multilocation learning, multikernel learning, multifeature protection, deep convolution encoder (DCE), bidirectional long short-term memory (BiLSTM), bearing fault diagnosis scheme

## Abstract

Considering various fault states under severe working conditions, the comprehensive feature extraction from the raw vibration signal is still a challenge for the diagnosis task of rolling bearing. To deal with strong coupling and high nonlinearity of the vibration signal, this article proposes a novel multilocation and multikernel scale learning framework based on deep convolution encoder (DCE) and bidirectional long short-term memory network (BiLSTM). The procedure of the proposed method using a cascade structure is developed in three stages. In the first stage, each parallel branch of the multifeature learning combines the skip connection and the DCE, and uses different size kernels. The multifeature learning network can automatically extract and fuse global and local features from different network depths and time scales of the raw vibration signal. In the second stage, the BiLSTM as the feature protection network is designed to employ the internal calculating data of the forward propagation and backward propagation at the same network propagation node. The feature protection network is used for further mining sensitive and complementary features. In the third stage of bearing diagnosis, the classifier identifies the fault types. Consequently, the proposed network scheme can perform well in generalization capability. The performance of the proposed method is verified on the two kinds of bearing datasets. The diagnostic results demonstrate that the proposed method can diagnose multiple fault types more accurately. Also, the method performs better in load and speed adaptation compared with other intelligent fault classification methods.

## 1. Introduction

Rolling bearings are widely used as indispensable components in modern mechanical equipment. However, the rolling bearings usually work under the severe conditions of varying speed, heavy load, variable load, and high temperature for a long time. They are vulnerable to occur deformation, abrasive wear, or other faults. These faults may lead to equipment performance degradation and even lead to severe economic loss [1]. Therefore, it is critically important to develop a system that can accurately diagnose various bearing faults under complex operating conditions and working environments.

From the perspective of pattern recognition, an intelligent bearing diagnosis process based on machine learning generally include three steps: data preparation, feature extraction, and fault classification. The purpose of feature extraction is to mining or summarize representative features. This operation can present the health condition of hardware devices and is beneficial to improve the accuracy of downstream fault classification tasks. Traditional bearing fault classification methods are difficult to extract features from the raw input signals, such as empirical mode decomposition [2], local mean decomposition [3], wavelet transform [4], Hilbert–Huang transform [5], etc. The aforementioned signal processing methods can obtain fault features of the different levels. Then, the extracted fault features with different input types are fed into the shallow machine learning model to obtain the diagnosis results, such as support vector machine [6], random forest [7], or logistic regression [8]. However, the upper-bound performances of machine learning algorithms are closely linked with the quality of feature mining or representation. The traditional intelligent fault diagnosis model composed of shallow learning approaches and feature extraction methods performs the following disadvantages.

- All features are naturally hand-crafted. The process of feature extraction requires much prior knowledge about diagnostic experience and signal processing technology, which needs to consume much labor and time resources. Complex and sophisticated modern equipment is difficult to extract the comprehensive and detailed internal features of rolling bearings.
- The feature extraction and fault classification of the diagnostic system are separately designed and performed, both of which impact the final classification result. However, the strategy cannot be optimized simultaneously.
- The limited inductive feature ability of shallow learning models cannot flexibly identify the complex state changes of the bearing. Fault diagnosis methods of the specific domain cannot be applied to other engineering fields. Therefore, a general-purpose method is needed to extend to new application areas.

Given the above drawbacks, deep learning (DL) combining feature extraction and fault classification may provide an effective solution for the bearing fault diagnosis system. In recent years, DL has made great achievements in many application fields such as face recognition, speech recognition, and computer vision. Schlemper et al. [9] utilized deep convolutional neural networks to reconstruct cardiac magnetic resonance images that are capable of preserving anatomical structure more faithfully. Ya et al. [10] solved the problem of face recognition across ages with deep learning. Motivated by these achievements, DL has achieved good performance in feature extraction and fault classification for the diagnosis system. DL attempts to construct the high-level representations of the input data using the multi-layer nonlinear processing unit in the hierarchical structure [11]. Because of the robust capabilities of extracting and adapting, DL can well establish a nonlinear mapping relationship between input data and pattern recognition. Compared with traditional intelligent fault diagnosis methods, the DL network has great performance in feature extraction and fault classification. Common examples of these DL methods include deep belief network [12], convolutional neural network [13], long and short-term memory neural network (LSTM) [14], deep convolutional autoencoder (DCAE) [15], etc.

The purpose of this article is to design an end-to-end bearing fault diagnosis system based on a deep convolutional encoder (DCE) and bidirectional LSTM (BiLSTM). The framework is motivated by their strong feature extraction abilities and classification effects. In essence, the DCE network is a cascade of a series of convolution neural network modules in structure. In the diagnostic field of rotating machinery, the equipment works in harsh environments and complicated working conditions. Thus, vibration signals of a rolling bearing are nonlinear and nonstationary caused by varying working conditions, along with much information irrelevant to fault diagnosis. From the mathematical viewpoint, the DCE may provide a novel solution to the bearing fault classification problem. DCE [16] can deeply compress and restore all the features of the input signal. Guo et al. [17] proposed a new DCE feature recognition framework, which successfully achieved the classification of multiple fault states of gearboxes. The encoder behaves like a filter and can help extract sensitive feature information through the deep network level to level and remove noise in the vibration signal. Therefore, we believe that DCE is more suitable for fault diagnosis of the rotating machinery compared with other intelligent methods. Due to its unique feature learning ability, DCE has been universally applied in fault diagnosis of gearboxes [18], bearings [19,20], and other rotating machinery [21].

Besides, LSTM has the advantage of dealing with nonlinear and long-term dependent dynamic problems in sequence data [22]. It can deeply mine the information correlation between vibration signals with similar features. The advantage is beneficial to the fault diagnosis of rolling bearings. The structure of BiLSTM can simultaneously utilize the information of past moments and future moments, which makes the final prediction more accurate than LSTM. An et al. [23] utilized CNN-based LSTM for fault feature extraction of the bearing under time-varying working conditions. Rao et al. [24] utilized convolutional BiLSTM to accurately realize fault diagnosis of rotating machinery. The abovementioned studies proved that DCE and BiLSTM have better diagnostic results than the normal machine learning networks in the fault diagnosis of rotating machinery. Therefore, this article combines DCE and BiLSTM as a basic network for comprehensive feature extraction of bearing fault information. Generally, in the diagnosis system, the features extraction by a single network are directly fed into the classifier. However, this approach may cause inaccurate or even loss of fault feature, resulting in a weakened classification effect of the diagnostic system under the complex working environment. Directly applying the present DCE-based and LSTM-based methods is challenging and improper for the bearing fault diagnosis. Thus, we intend to analyze the dilemma from four aspects.

In the first aspect, the rolling bearing usually works in varying operation conditions, especially under varying speeds and loads. On this account, the nonlinear vibration signal measured by the sensor is commonly coupled and complex [25]. If the input signal is directly sent to a neural network, the traditional methods with time-space processing will lose the detailed information in the frequency space. We know that deep networks are specialized in processing highly nonlinear data, so we use the multiscale wavelet transform technology to map the raw vibration signals to the wavelet domain for deeper understanding and mining.

In the second aspect, there is only one next layer structure for network propagation in the DL architecture, and the network outcome only contains the fault feature of the last layer in the feature extraction process. As the network deepens, these features will be more robust. Although traditional network frameworks can extract some robust and invariant features for bearing fault diagnosis, these features will lose some precise and detailed information that distinguishes the varying fault conditions. Known from the field of image recognition [26], CNN can directly learn abstract and robust features from two-dimensional and higher-dimensional images. Each CNN integration module can only extract local features of vibration signals. The structure of DCE is equivalent to the cascade of multiple CNN integration modules, and each module alternately convolutes and pools on the maps to perceive the local characteristics of the vibration signal. Thus, it is inadequate to use only the features directly extracted from the last layer. Inspired by the above research, we propose the multilocation scale learning framework. In each kernel branch, the proposed framework connects a certain skip layer to the last output layer of the encoder. The positions of the skip connection can skillfully choose from different CNN integration modules. Therefore, the skip layer allows multilocation feature learning to dig out more comprehensive features from the input. Then, the multifeature fusion technology is employed to fuse and optimize the signal features learned from multiple learning branches. Therefore, the proposed network can effectively complete complex classification tasks.

In the third aspect, it is well known that rolling bearings are an important component of mechanical systems. There are many interactional and coupling effects among rolling bearings and other mechanical components. Vibration signals measured from rolling bearings commonly contain complex signals of various mechanical vibrations. Vibration signals usually exhibit multiscale properties [27]. However, the traditional DCE has a poor ability to capture multiscale features. To overcome the limitation, this article introduces feature learning with multiple kernel scales into the encoder. Each CNN integration module with different convolution kernels is employed to extract multiscale features layer by layer, and the fault features are refined and compressed. Based on these modules, the multiscale learning network is proposed to mining deeper and comprehensive fault features from the vibration signal. Therefore, the work can enhance the robustness of encoder fault feature learning. Thus, combining the skip connection and multiple kernel branches, we propose a strong network scheme of fault diagnosis with multilocation and multikernel scale learning defined as the generalized multiscale learning (GMSL).

In the fourth aspect, DL algorithms generally employ dropout coefficients in network propagation to prevent network overfitting. The operation may weaken the importance of some features or lose the integrity of some features. To overcome the difficulty, the multifeature protection layer is introduced into the fault diagnosis system and forms a series structure behind the GMSL fusion layer. Vibration signal is a time series signal with the attribute of data dependence. The bidirectional long and short-term memory network with feature protection (PBiLSTM) is designed after the GMSL network. Based on the robust features extracted by the GMSL network, PBiLSTM considers the relationship between current and future information to extract data-dependent features. Meanwhile, PBiLSTM employs the internal calculating data of the forward propagation and backward propagation to dig more sensitive features at the same network position. This PBiLSTM network is, to a certain extent, the integration and protection of multifeature fault signals.

Combining the above four innovations, in this article, a framework scheme with multikernel scale learning and multilocation scale learning cascading the feature protection layer (MLKDCE-PBiLSTM) is proposed. The scheme can adaptively extract and fuse bearing fault features from multiple network locations and time scales of the raw data.

The main contributions of this article are summarized as follows:

1. This article combines the skip connection and encoder network and proposes a multilocation scale learning network that extracts global and local features from the network layers of different depths. The advantages of this feature extraction can be accumulated in the entire network by adding multiple skip connections.
2. Multikernel scale learning is introduced into the CNN integration module of the DL with different kernel sizes to simultaneously learn vibration characteristics at the different time scales. The advantages will be accumulated in the entire network by adding multiple kernel scale branches.
3. The feature information fusion layer is employed to automatically fuse the feature space and optimize the rich features extracted from the multilocation learning network and multiscale learning network.
4. The PBiLSTM network is used to deeply excavate the efferent robustness features of the GMSL network and captures dependent and sensitive fault features.
5. Based on the above improvements, the MLKDCE-PBiLSTM scheme is proposed to extract comprehensive fault features. The MLKDCE-based network can autonomously extract and fuse useful and comprehensive features using multilocation and multiscale learning. However, the PBiLSTM-based network is designed to deeply excavate and protect high-purity features of GMSL network output. Consequently, under the complicated working conditions of varying speeds and loads, the proposed feature learning method is used to accurately diagnose various fault types of rolling bearings.

The progress of this article is organized as follows. Section 2 introduces the theoretical background. Section 3 elaborates the network scheme of this article. Section 4 uses two kinds of data sets to evaluate the above method. Section 5 verifies the functions of each component of the proposed model. Finally, Section 6 summarizes the whole article.

## 2. Theoretical Background

### 2.1. Multiscale Wavelet Transform (MSWT)

**Figure 1:**
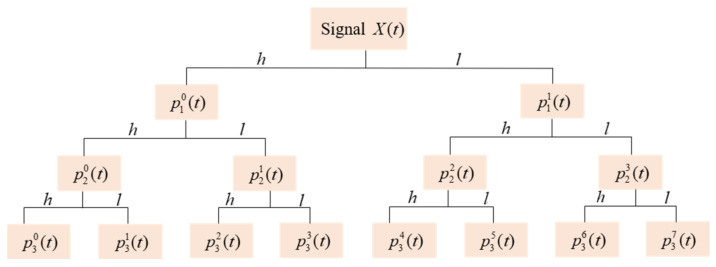
Illustration of Multiscale Wavelet Transform.

Various methods of transforming and extracting features of the original data are used for fault diagnosis of bearings. MSWT can observe the signal gradually from coarse to fine with the multiscale characteristics, which is adjustable for various frequencies in the time domain sampling step. The rules of slow changes under low-frequency signals and rapid changes under high-frequency signals have better effects for diagnosis tasks. MSWT overcomes the inflexible time-frequency window characteristics of Fourier transform. In short, using low-pass filter (LPF) h(k) and high-pass filter (HPF) l(k), the vibration signal *X(t)* is iteratively decomposed into [28,29].

(1) $$p_{i+1}^{2n}\left(\tau \right)=\underset{k}{\sum }h\left(k-2\tau \right)p_{i}^{n}\left(k\right)$$

(2) $$p_{i+1}^{2n+1}\left(\tau \right)=\underset{k}{\sum }l\left(k-2\tau \right)p_{i}^{n}\left(k\right)$$

where the signal *X(t)* is { $p_{0}^{1}\left(k\right),k=1,2,\ldots ,N$ } (*N* is the raw vibration signal length). At the *i*th level, $p_{i}^{n}\left(k\right)$ denotes the MSWT decomposition coefficients of the *n*th. At (*i+1*)th level, $p_{i+1}^{2n}\left(\tau \right)$ refers to the approximation coefficients and precise coefficients of LPF and HPF of the *2n*th; $p_{i+1}^{2n+1}\left(\tau \right)$ refers to the coefficients of *(2n+1)*th nodes. Therefore, the full vibration signal can be divided into a multiscale frequency band for the precise signal analysis by MSWT, which is achieved through a recurrent filter of LPF and HPF. At the *i*th layer, *X(t)* is decomposed into $2^{i}$ nodes, which are denoted as (*i, n*) ($n=0,1,2,\ldots ,2^{i-1}$) in the binary decomposition tree. As shown in Figure 1, *X(t)* can be decomposed into eight different time-frequency subspaces by a three-level MSWT.

**Figure 2:**
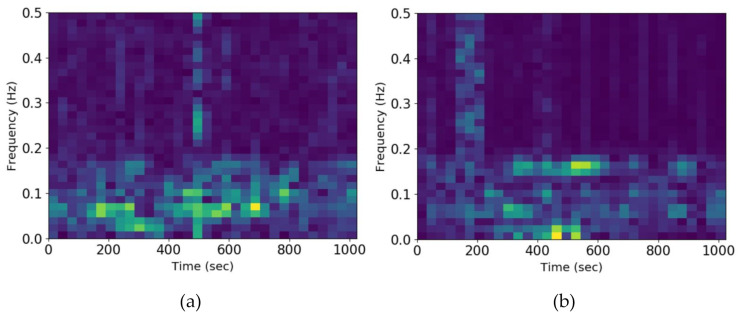
Scalogram of time and frequency under different datasets. (**a**) Scalogram in PU dataset; (**b**) Scalogram in CWRU dataset.

Contrary to the operation of aforementioned recursive split in Formulas (1) and (2), and the reconstruction process based on MSWT coefficients can be expressed as

(3) $$p_{i}^{n}\left(\tau \right)=\underset{k}{\sum }H\left(k-2\tau \right){\tilde{p}}_{i+1}^{2n}\left(k\right)+\underset{k}{\sum }L\left(k-2\tau \right){\tilde{p}}_{i+1}^{2n+1}\left(k\right)$$

where $\tilde{p}$ means to insert a zero beside each point of *p*. To reconstruct signals of the same length as *X(t)*, except for the (*i, n*) node, it is necessary to set all the coefficients of the other nodes in the *i*th reconstruction node to zero. This is just to preserve the frequency information of reconstructed nodes. In Figure 2, two load frequency distribution is revealed by the analytical method of time and frequency. For each scalogram, the horizontal axis stands for a time distribution, while the vertical axis is a frequency distribution under a specific representation. The vibration signals have different frequencies at different times. Also, vibration signals have different frequencies at the same moment. The frequency distribution of different loads shows different brightness. 

### 2.2. Activation Function

**Figure 3:**
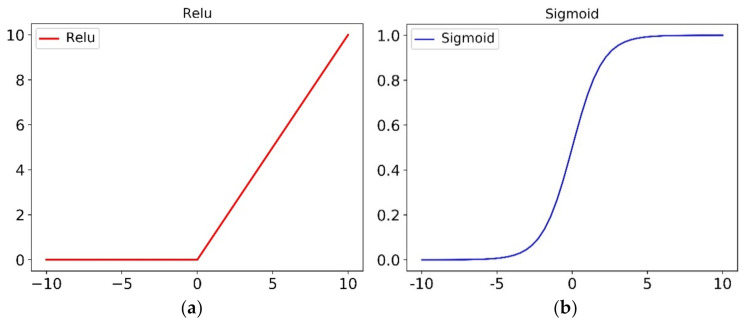

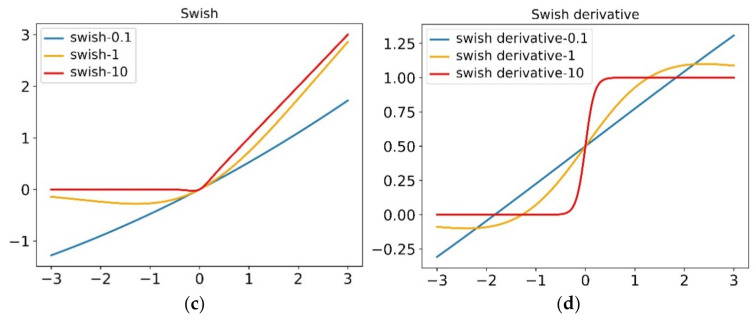
Four curve diagrams of activation function. (**a**) Relu activation function, (**b**) Sigmoid activation function, (**c**) Swish activation function under various learnable parameters, (**d**) Swish derivative under various learnable parameters.

As the most common nonlinear unit of deep learning activation function, the choice of activation function in a deep network has a great impact on the training process and classification result. Swish is a new self-gated activation function. The researchers conducted experiments on multiple complicated datasets and proved that the Swish activation function is better than ReLU on the deep model [30]. It is simplicial and similar for the Swish compared with the ReLU. The advantage allows us to easily replace with Swish function in the network propagation. The Swish and its derivative are shown in Figure 3. The Swish activation function expression is

(4) $$Swish\;\left(x\right)=x*\alpha \left(x\right)$$

(5) $$\alpha \left(x\right)=\frac{1}{1+e^{-x}}$$

The Swish derivative is

(6) $$Swish^{\prime }\left(x\right)=\sigma \left(x\right)+x*\sigma \left(x\right)\left(1-\sigma \left(x\right)\right)\;=f\left(x\right)+\sigma \left(x\right)\left(1-f\left(x\right)\right)$$

The Swish activation function has the following advantages.

1. The functions have three characteristics of lower bounds, no upper bounds, and non-monotonic.
2. Both Swish and its first derivative have smooth characteristics.

In this article, all CNN integration modules employ the activation function Swish. 

### 2.3. Deep Convolutional Autoencoder (DCAE)

Autoencoder and PCA are similar, both of them can dimensionality reduction and feature extraction for data. However, among the autoencoder, PCA, and kernel PCA, there are lots of differences. They are summarized as follows:

1. In the coding process, the autoencoder can perform both linear transformations with a linear activation function and a nonlinear transformation with a nonlinear activation function. When PCA performs a nonlinear data process, it is assumed that the data conform to ideal data distribution. Otherwise, PCA can only perform linear transformations [31].
2. In this article, the input data is processed into an image by the Wavelet Transform. The bearing dataset is highly nonlinear and complicated. For the autoencoder, it can learn the linear and nonlinear features with encoder and decoder. However, PCA can only learn the linear features.
3. The dimensions of the kernel PCA method are dependent on the number of input data in the eigen-decomposition. The autoencoder is flexible. In structure construction, because of the network representation form of an autoencoder, multiple nonlinear layers can be used for feature extraction.

The autoencoder has more advantages compared with PCA.

- The structure of the autoencoder is much more flexible than PCA, which can process more diversified vibration data.
- The application of autoencoder is wider, such as data denoising, visualization and dimension reduction, image compression, and feature learning.
- PCA is just a special case of a single-layer autoencoder with a linear activation function.

**Figure 4:**
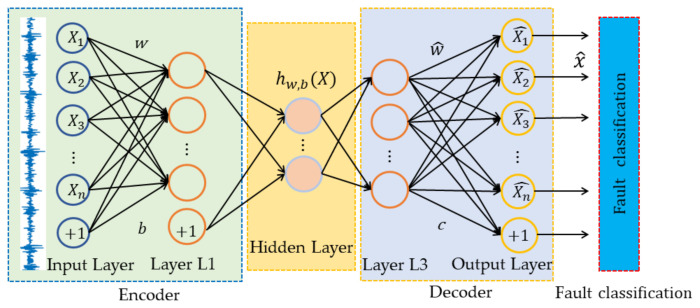
Architecture of a DCAE with three feature extraction layers.

The architecture of DCAE consists of two parts: encoder and decoder, which can be seen as the combinations of convolution layer, pooling layer, deconvolution layer, and unpooling layer. This architecture employs the backpropagation theory to extract the key feature information and expresses the information through feature compression, which well stains spatial information of the 2D signal. The encoder is a neural network model that can learn and discover the hidden features of the input data. The decoder is a neural network model that can reconstruct the original input data using highly compressed features of hidden layers. Figure 4 shows a three-layer DCAE model.

Suppose we have k convolution kernels, and each convolution kernel is composed of parameters $w^{k}$ and $b^{k}$. The parameters are used to express the convolutional layer, then the obtained feature $h^{k}$ is used to reconstruct. The following formula can be obtained

(7) $$h^{k}=\sigma \left(x*w^{k}+b^{k}\right)$$

(8) $$\hat{x_{i}}=\sigma \left(h^{k}*{\hat{w}}^{k}+c^{k}\right)$$

The error *Loss* is obtained by comparing the input sample and feature reconstruction result with Euclidean distance, which is optimized with the BP algorithm. The error of the DCAE is expressed as

(9) $$Loss=\frac{1}{2n}\overset{2n}{\underset{1}{\sum }}{\left(x_{i}-\hat{x_{i}}\right)}^{2}$$

where $h^{k}$ represents the convolution value of the encoder, and $\hat{x}$ represents the reconstruction value of the raw data. $w^{k}$ and ${\hat{w}}^{k}$ represent the weight of the encoder and decoder, respectively. Similarly, $b^{k}$ and $c^{k}$ are the corresponding bias parameters, and $\sigma \left(\cdot \right)$ is the activation function. *Loss* represents the loss of each weight w.

DCAE is composed of multiple convolutional neural networks, so the encoder of DCAE is designed to extract and compress features of the input signal level to level. The purpose of the fault diagnosis system is to obtain high-purity discriminative features, so we only introduce the encoder to the fault diagnosis system. Generally, the architecture of a convolutional encoder is regarded as an integration of a feature learning layer, a nonlinear transform layer, a normalization layer, and a feature pooling layer. At the feature learning layer, the input of each unit is connected to the output of the previous module, and the local features are extracted by the convolution kernel. In the nonlinear activation layer, the features of the lower dimension are mapped to the higher dimension space by selected activation functions. This convolution function can be expressed as

(10) $$V_{n}^{i,k}=\sigma \left(\sum_{m}{Χ}_{m}^{i-1}⊙K_{n}^{i}+b_{n}^{i}\right)$$

where ${Χ}_{m}^{i-1}$ and $V_{n}^{i,k}$ respectively represent feature vectors of the *m*th input and *n*th output at (*i-1*)th layer in the feature extraction. $K_{n}^{i}$ represents the convolution kernels between the *m*th input nonlinear transform and the *n*th output nonlinear transform and *k* expresses the sum of convolution kernels. $b_{n}^{i}$ presents the deviation of the *n*th output nonlinear transform, and $\sigma \left(\cdot \right)$ is the nonlinear activation function.

Multikernel branch of the proposed MLKDCE-PBiLSTM employ convolutional encoders. The specific operations are described in Section 3.

### 2.4. Bidirectional Long Short-Term Memory Network

The standard RNNs (time recurrent neural networks) structure is a chain form of repeated neural network modules, and a directed connection is established through mathematical relationships. Different from the basic model structural multilayer perceptron, RNNs can map the target vector from the entire input history input, while the multilayer perceptron can only map from the original input to the target vector. Due to the characteristics of RNNs that allow historical states to be kept in the memory of the network, for supervised learning, RNNs can be repeatedly trained through backpropagation. Due to the characteristics of RNNs that allow historical states to be retained in the memory of the network, for supervised learning, RNNs can be repeatedly trained through backpropagation. To capture the semantics in the long sequence, we need to run the RNN on multiple time steps and turn the unrolled RNN into a deeper network. However, this method brings about the gradient vanishing situation as RNN training, which seriously affected the accuracy of the fault classification. This means that traditional RNNs may not be able to capture long-term dependencies.

**Figure 5:**
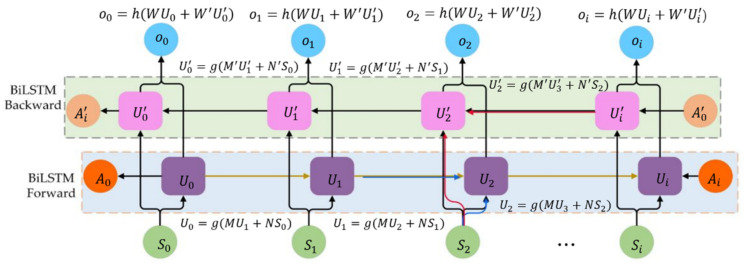
Architecture of the BiLSTM network for the sequential data.

The emergence of LSTMs solves the above gradient vanishing and gradient explosion problems. In LSTMs, memory units including input gates, forget gates, and output gates replace each neuron in hidden layers of RNN. In each component, input gates update the unit states; forget gates selectively discard certain information and reset memory units to prevent the long-term dependence; output gates output unit states. For capturing the valid dynamic characteristics of nonlinear time series data, LSTMs perform more advantages than traditional CNN. LSTMs have been successfully used in speech recognition, natural language processing, subtitle translation, picture description, and many other occasions. The MLKDCE-PBiLSTM in this article employs PBiLSTM to build a time series model. PBiLSTM consists of two ordinary RNNs, a forward RNN that uses past information, and a backward RNN that uses future information. The network diagnoses more accurately than the prediction result obtained by using LSTM alone. When extracting fault features at time t, each unit gate can simultaneously apply the calculated data at t−1 and t+1 time. The structure of PBiLSTM is illustrated in Figure 5.

$U_{0}\to U_{1}\to \cdots U_{i}$ represents the forward RNN, which participates in the forward calculation. Specially, the input value at time t is the algebraic sum: the sequence data $S_{t}$ at time t and the output value $U_{t-1}$ at time t−1.

$U_{i}^{\prime }\to \cdots U_{2}^{\prime }\to U_{0}^{\prime }$ represents the backward RNN, which participates in the backward calculation. Specially, the input value at time t is the algebraic sum: the sequence data $S_{t}$ at time t and the output value $U_{t+1}^{\prime }$ at time t+1.

The final output data at time *t* depends on $U_{t-1}$ and $U_{t+1}^{\prime }$.

## 3. Comprehensive Feature Learning Method

**Figure 6:**
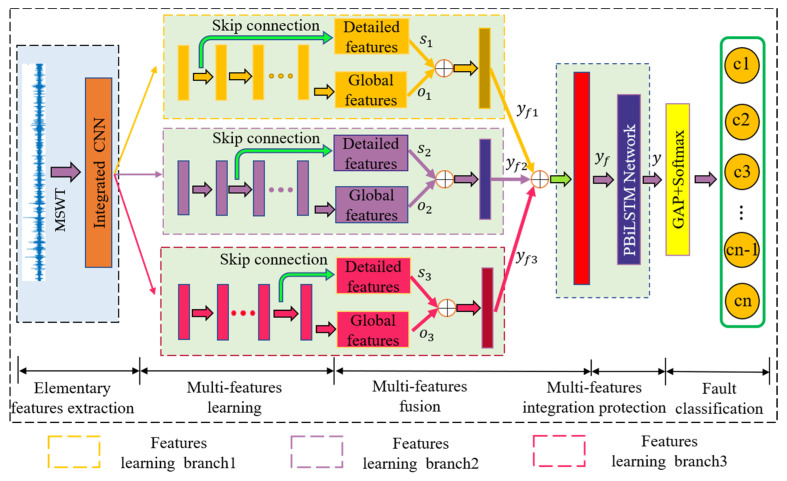
Architecture of the proposed MLKDCE-PBiLSTM scheme.

This article intends to provide a network scheme that can automatically learn the multifeature from various time scales of the input data by the varying skip connections and the multiscale learning. The operation of fusing the feature space can improve the fault diagnosis performance of rolling bearings under variable conditions of loads and speeds. Especially, the multifeature protection layer is cascaded to the GMSL network of the fault diagnosis system. A reliable intelligent fault diagnosis system should accomplish comprehensive and in-depth feature extraction of the vibration signal, and simultaneously performs global and local features learning. The core contribution of this article is to construct an end-to-end framework that integrates feature transformation, multiscale learning, multifeature fusion, multifeature protection, and fault classification. The network combines multiscale learning and multifeature protection to comprehensively and deeply extract the signal feature. The overall MLKDCE-PBiLSTM framework of bearing fault diagnosis is shown in Figure 6. The first step is to perform wavelet processing on the raw signal to construct a 2D image. Then 2D images as network input are fed into the elementary features extraction layer to perform the feature extraction at the initial period. In this process, a bigger convolution kernel is employed to ensure that the features of the input data are completely extracted. In this article, multiscale feature learning has two meanings, one is multilocation scale learning with multiple skip connections, and the other is multikernel scale learning with different kernel sizes under multiple branches. Similarly, multifeature fusion refers to fuse the skip layer and the last layer of convolution in each kernel branch on the one hand and fuse the GMSL features of the MLKDCE-PBiLSTM framework on the other hand.

The fusion features of GMSL are relatively pure (filtering operation of multiscale convolutional neural network). The features are fed into the feature protection module for sensitive and ultimate feature extraction that all features are re-extracted by the network and given more reasonable weight coefficients at this time. In this case, it is more conducive for PBiLSTM to extract new and in-deep discriminative features by considering the front and back sequential relationship of the fault signal. Finally, the newly extracted features are fed into the softmax layer, so that the probability distribution of each sample is clearly obtained. Thus, the multiscale features extracted of MLKDCE-PBiLSTM are much more robust with precise details, which effectively realizes the task of feature recognition and fault classification.

### 3.1. Generalized Multiscale Learning (GMSL)

The bearing fault diagnosis method with multilocation scale learning and multikernel scale learning is defined as the generalized multiscale learning. The method has been verified to be much more robust in this article.

#### 3.1.1. Multilocation Scale Module (MLS)

In the academic research of diagnosis task, only the single layer of the convolutional encoder network is commonly employed for the next layer input in network propagation, and the network outcome only contains the fault information of the last layer in the feature extraction process. Although the deep features extracted from the last layer of multilocation feature learning (MLFL) are more invariant and robust than the features of the lower layers, the multilayer convolution operation may lose many sensitive and detailed features that exist in the middle layer. Therefore, it is insufficient to directly use the features extracted from the last layer.

In each branch, MLS considers the feature mapping which contains a certain middle layer of the network and the last layer of the network by a skip connection as the input of the feature fusion layer. Therefore, the network can learn the fault features of different convolutional layers, effectively combining the MLFL with the convolutional encoder. The core of MLFL is to learn invariant features (global feature) and detailed features (local feature) of vibration signals in different network locations. So better classification performance can be achieved. Specifically, MLFL uses skip connections in the multilayer convolutional structure to select the middle layer (one or more) of the network at different locations to combine with the final convolutional layer. The network can simultaneously learn the discriminative fault features of different locations and adopts the convolution operation to fuse multilocation features across channels.

**Figure 7:**
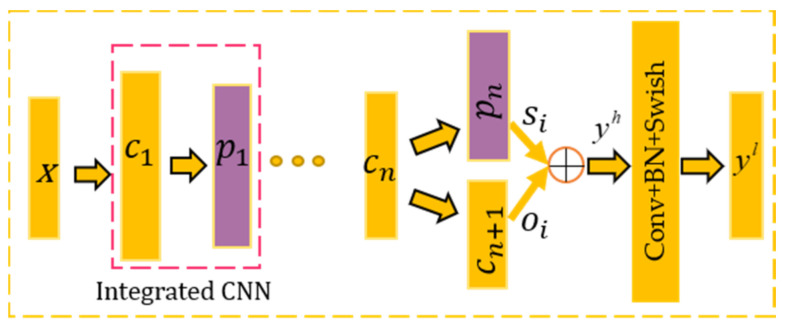
Structure of the MLFL.

In the illustration, we only use the penultimate convolutional layer as skip layer for the limited computing power. According to Figure 7, MLFL uses n serial CNN integration modules with convolution layer and maxpool layer to learn the rich features of the input signal *X(t)* at different locations in the network. The output of MLFL fusion layer is expressed as

(11) $$y_{j}{}^{l}=\sigma \left(\underset{i}{\sum }h_{i}^{c}⊙w_{i,j}^{c}+\underset{i}{\sum }h_{i}^{s}⊙w_{i,j}^{s}+b_{j}\right)$$

where ⊙ represents convolution operation, *i* denotes the *i*th feature map of the (*n-1*)th convolutional layer. *j* denotes the *j*th feature map of the *n*th largest pooling layer, $h_{i}^{c}$, $w_{i,j}^{c}$, $h_{i}^{s}$, and $w_{i,j}^{s}$ represent neurons and network weights of the (*n-1*)th layer and the *n*th maxpool layer, respectively. BN presents batch normalization (BN) [32].

First, $y^{h}$ performs feature fusion by the feature fusion layer $C_{l}$. Then, $y^{h}$ is fed into the CNN integration module for deep feature mining again. Finally, the MLFL output layer is $y^{l}=\sigma \left(C_{l}\sum y_{j}^{h}⊙\omega_{j}^{h}\right)$.

#### 3.1.2. Multikernel Scale Module (MKS)

The core of multikernel feature learning (MKFL) is to learn the multiscale complementary features of vibration signals in different time scales. MKS can skillfully adjust the size of the convolution kernel to enable CNN integration modules to extract the fault characteristics of different time scales. The module successfully combines the MKFL with the encoder. Specifically, MKFL uses several parallel network branches, that is, each CNN integration module in the multiple branches has different sizes of the convolution kernel. Therefore, the network can learn the rich vibration features of different scales at the same time, and use the convolution operation to fuse the features of multiple time scales across channels.

**Figure 8:**
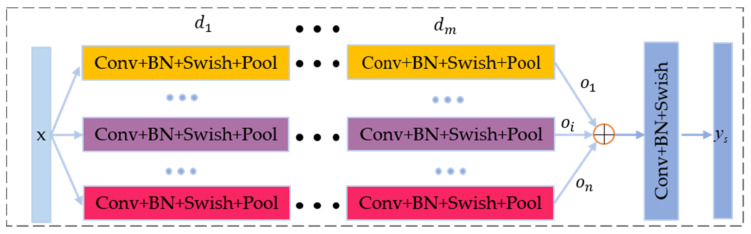
Structure of the MKFL.

According to Figure 8, MKFL uses n parallel CNN integration modules that consist of convolution layer and maxpool layer and m branches to learn the rich features of the vibration signal *X(t)*. The output of the MKFL layer of each branch is expressed as

(12) $$o_{i}=\overset{m}{\underset{1}{\cup }}(\sigma_{i}\left(\omega_{i}⊙x+b_{i}\right))$$

where $\cup_{1}^{m}\left(•\right)$ represents a continuous convolution operation, that is, convolution operations are performed on all the feature output layers in sequence.

The outputs of n MKFL branches are connected into feature vectors $O=\left[o_{1},o_{2},\ldots ,o_{n}\right]$ across channels. Then vectors O is put into the feature fusion layer $C_{s}$, which effectively fuses complementary features of various kernel branches. The output of the fusion layer is expressed as $y_{s}=C_{s}\left(O\right)$.

### 3.2. Multifeature Fusion

In this article, to give full play to the comprehensive capabilities of fault feature extraction, each network branch is designed to simultaneously embed the MLS module and MKS module. The combination is regarded as a new network module with comprehensive feature learning (CFL) module. This is more conducive to extract abundant and in-depth feature of the raw input signal. The output of each CFL branch network is $y^{h}=\left[s_{i},o_{i}\right]$, Using the fusion layer $C_{s}$, the GMSL can be expressed as $y_{f_{i}}=GMSL\left(x\right)=C_{s}\left(y^{h}\right)=C_{s}(\left[s_{i},o_{i}\right]$).

The proposed GMSL fuses the fault feature of multiple network locations and multiple time scales. Such deep features are much more abundant and complementary with precise details. However, these robust features may not promote each other, resulting in the features weakening. Therefore, it is necessary to use a valid feature fusion mechanism for the multikernel structure. The features of multiple CEL branch network are the different levels of understanding for the raw signal.

In the following two aspects, MLKDCE uses the multifeature fusion method. One is to employ the feature fusion layer $C_{l}$ to fuse the multilocation features learned from MLS. The other is to employ the feature fusion layer $C_{s}$ to fuse the MKS features learned from MKS. Both $C_{l}$ and $C_{s}$ adopt the feature learning and nonlinear transformation of different depth convolutional layers to adaptively integrate multifeature signal; the main difference is the location of the skip connection in each branch and the kernel size of the feature extraction. According to Figure 6, the MLKDCE-PBiLSTM concatenates $y_{f_{1}}$,$\;y_{f_{2}}$ and $y_{f3}$ of the multifeature learning into the feature vector $y^{F}=\left[y_{f_{1}},y_{f_{2}},y_{f3}\right]$. Then multifeature fusion layer $C_{s}$ adaptively fuse the abundant feature vector $y^{F}$. The final feature learned from the raw vibration signal *X(t)* can be obtained by $y_{f}=C_{s}\left(y^{F}\right)=C_{s}\left(\left[y_{f_{1}},y_{f_{2}},y_{f3}\right]\right)$. This CFL method helps to provide excellent classification effect for bearing fault diagnosis tasks.

### 3.3. Multifeature Protection Layer

In the bearing fault diagnosis system, the fused multifeature vector is directly put to the classification layer. The operation may weaken the importance of some features or even lose the integrity of some features, and may not performs the best classification effect. Therefore, it is necessary to use valid feature integration and protection mechanisms. The basic LSTM network is limited in its ability to effectively use the context. In the process of bearing fault feature learning, there is a strong dependence between sequential perception data. Considering the connection between current and future data is a kind of protection for feature integrity. PBiLSTM has achieved remarkable success in feature extraction of dependent sequence data [33,34]. Therefore, this article introduces PBiLSTM into the protection layer for the feature output of the multikernel networks.

PBiLSTM can process the sequence data forward and backward through two bidirectional units which are fed forward to the same output layer [35].

(13) $$\vec{i^{t}}=\sigma \left(\vec{W^{i}}⊙\vec{y_{f}{}^{t}}+\vec{V^{i}}⊙\vec{h^{t-1}}+\vec{b^{i}}\right)$$

(14) $$\vec{f^{t}}=\sigma \left(\vec{W^{f}}⊙\vec{y_{f}{}^{f}}+\vec{V^{f}}⊙\vec{h^{t-1}}+\vec{b^{f}}\right)$$

(15) $$\vec{o^{t}}=\sigma \left(\vec{W^{o}}⊙\vec{y_{f}{}^{o}}+\vec{V^{o}}⊙\vec{h^{t-1}}+\vec{b^{o}}\right)$$

(16) $$\vec{c^{t}}=\vec{f^{t}}*\vec{c^{t-1}}+\vec{i^{t}}*tanh\left(\vec{W^{c}}⊙\vec{y_{f}{}^{t}}+\vec{V^{c}}⊙\vec{h^{t-1}}+\vec{b^{c}}\right)$$

(17) $$\vec{h^{t}}=\vec{o^{t}}*tanh\left(\vec{c^{t}}\right)$$

(18) $$\overset{\leftarrow }{i^{t}}=\sigma \left(\overset{\leftarrow }{W^{i}}⊙\overset{\leftarrow }{y_{f}{}^{t}}+\overset{\leftarrow }{V^{i}}⊙\overset{\leftarrow }{h^{t-1}}+\overset{\leftarrow }{b^{i}}\right)$$

(19) $$\overset{\leftarrow }{f^{t}}=\sigma \left(\overset{\leftarrow }{W^{f}}⊙\overset{\leftarrow }{y_{f}{}^{f}}+\overset{\leftarrow }{V^{f}}⊙\overset{\leftarrow }{h^{t-1}}+\overset{\leftarrow }{b^{f}}\right)$$

(20) $$\overset{\leftarrow }{o^{t}}=\sigma \left(\overset{\leftarrow }{W^{o}}⊙\overset{\leftarrow }{y_{f}{}^{o}}+\overset{\leftarrow }{V^{o}}⊙\overset{\leftarrow }{h^{t-1}}+\overset{\leftarrow }{b^{o}}\right)$$

(21) $$\overset{\leftarrow }{c^{t}}=\overset{\leftarrow }{f^{t}}*\overset{\leftarrow }{c^{t-1}}+\overset{\leftarrow }{i^{t}}*tanh\left(\overset{\leftarrow }{W^{c}}⊙\overset{\leftarrow }{y_{f}{}^{t}}+\overset{\leftarrow }{V^{c}}⊙\overset{\leftarrow }{h^{t-1}}+\overset{\leftarrow }{b^{c}}\right)$$

(22) $$\overset{\leftarrow }{h^{t}}=\overset{\leftarrow }{o^{t}}*tanh\left(\overset{\leftarrow }{c^{t}}\right)$$

Then, the representation of complete PBiLSTM hidden unit $h^{t}$ is a cascaded vector output from the forward and backward processes, the formula is

(23) $$h^{t}=\vec{h^{t}}⊕\overset{\leftarrow }{h^{t}}$$

### 3.4. Fault Classification

In this article, the classification task of rolling bearing is a multi-classification task. Generally, the features obtained by the last layer of the traditional BiLSTM network are vectorized and then sent to the conventional fully connected layer and feature classifier. This approach makes the parameters of the fully connected layer very large and prone to overfitting. Thus, in the output layer, we use the global average pooling layer (GAP) instead of the fully connected layer (FCL) [36] and output the conditional probability for each class by the softmax function. One of the benefits of this operation is that the feature map is directly related to the diagnosis accuracy. Another advantage is that the GAP does not need to calculate and optimize additional network parameters. Therefore, the model scale and calculation are greatly reduced compared with the FCL, and overfitting can be prevented.

It is assumed that there are n types of input samples, and the output probability $Q_{j}$ of *k*th class is calculated as (24). The diagnostic output is the fault label corresponding to the maximum $Q_{j}$.

(24) $$Q_{j}=\frac{exp\left(\theta^{\left(j\right)}GAP\left(y\right)\right)}{\sum_{j=1}^{n}exp\left(\theta^{\left(j\right)}GAP\left(y\right)\right)},\;j=1,2,\ldots ,n$$

where $\theta^{\left(j\right)}$ denotes the network parameter; $GAP\left(y\right)$ expresses the input of the model and $\sum_{j=1}^{n}Q_{j}=1$.

For MLKDCE-PBiLSTM training, we use the cross-entropy as the loss function, which is the absolute value of the true class label and predicted class label. The Adam optimization algorithm [34] is adopted to minimize the loss value, which has high computational efficiency and less memory.

Meanwhile, the proposed MLKDCE-PBiLSTM scheme is general and flexible, which may have multiple network branches and different convolution depths in each branch. Specifically, each branch can have a different skip location and different convolution kernel scales. MLKDCE-PBiLSTM can effectively learn abundant and complementary diagnosis information at different time scales by using the skip connection structure in multiple branches. PBiLSTM can effectively capture abstract fault features by adopting the hierarchical learning framework in multiple branches.

## 4. Experimental Setup

In the bearing fault diagnosis, to verify the effectiveness of the MLKDCE-PBiLSTM in the complex feature extraction, this article conducts several experiments. The experimental data are the bearings datasets of Paderborn University (PU) and Case Western Reserve University (CWRU).

### 4.1. Description of PU Datasets

Lessmeier et al. provided a PU benchmark bearing dataset for bearing condition monitoring and diagnosis [37] and described the corresponding dataset in detail. A total of 32 bearings were used in the PU dataset: 12 bearings for artificial damage, 14 bearings for accelerated lifetime test, and 6 healthy bearings. All bearings were tested under four different test conditions, as shown in Table 1. The data is perpendicular to axis and frequency is 64 kHz. There are six main bearing damage modes: fatigue, wear, corrosion, electrical erosion, plastic deformation, fracture, and cracking. Besides, the bearing 6203 faults are divided into four damage levels to determine the extent of the damage. The first level represents the damage length is less than 2 mm, the second level represents the damage length greater than 2 mm, the third level represents the damage length greater than 4.5 mm, and N/a represents damage length greater than 13.5 mm. Finally, all the bearings are installed on a special test bench for data collection. The experiment collected five parameters: motor current and vibration signals, as well as load torque, radial force, and oil temperature.

**Figure 9:**
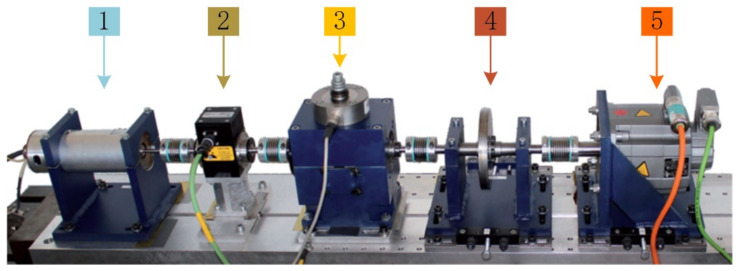
Test rig of experimental system: (**1**) test motor; (**2**) measuring shaft; (**3**) bearing module; (**4**) flywheel; (**5**) load motor.

All the bearings are stalled in the experiment system. The modular setup in the test rig is designed to collect the PU datasets in various load conditions. The platform is shown in Figure 9.

To simulate the varying working conditions of rolling bearing as much as possible, in the following case study, this article uses the real damaged datasets to study the fault diagnosis of the motor bearing. Under the four working conditions described in Table 2, four bearings with outer ring fault, four bearings with inner ring fault, and six bearings with mixed outer ring fault and inner ring fault are used. In this article, five bearing datasets are employed to validate the proposed model, shown in Table 3. The damage degree is also classified as four levels. There are 5600 samples for each health condition under each load (4800 trainings and 800 tests). All samples employ MSWT and data augmentation technology. Since MSWT is time-consuming, the length of each sample is set to 100.

### 4.2. Description of CWRU Datasets

**Figure 10:**
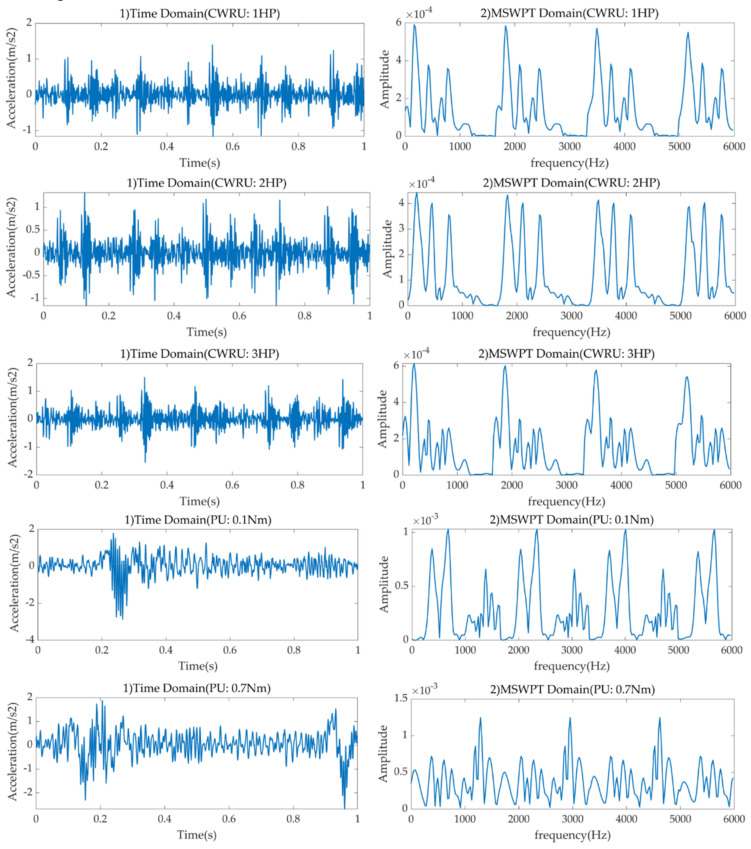
Visualization of signal from PU and CWRU in time domain and frequency domain under different loads.

According to the experimental requirements designed in the paper, four datasets with normal state, rolling element fault, inner raceway fault, and outer raceway fault are selected. Single point faults with sizes of 0.007, 0.014, and 0.021 are set on the four kinds of bearing drive-end fault types [38]. All bearing faults are processed by EDM technology. The vibration results are recorded at 12 kHz frequency under operating conditions of three sizes and four different horsepower (0, 1, 2, and 3 horsepower). The visualization of the signal in the time domain and frequency domain is shown in Figure 10. The test motor model is SKF6205-2RS, and its size parameters are shown in Table 4. In this article, five bearing datasets are employed to validate the proposed model, shown in Table 5. There are 5600 samples for each health condition under each load (4800 trainings and 800 tests). All samples employ MSWT and data augmentation technology before being input to the MLKDCE-PBiLSTM model. Since MSWT is time-consuming, the length of each sample is set to 100. A time-frequency image with a size of 100 × 100 will be generated.

### 4.3. Data Processing and Augmentation

The intelligent fault diagnosis system is an end-to-end learning method, but the types of vibration data and the methods of normalization have a great effect on its performance. The signals we collected are time series, which are the raw signals, and it generally performs poor results when are employed directly. A reasonable type of input signal is significant to the performance of the DL model. So MSWT methods and BN normalization methods are employed in this article to process the raw data, which is detailed in Section 2.1.

**Figure 11:**
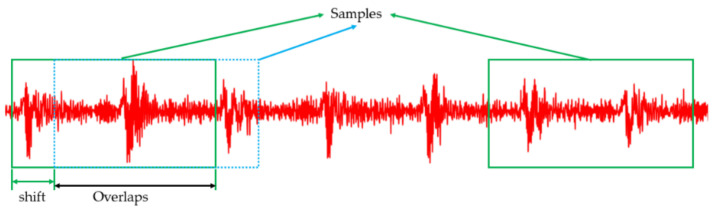
Data augmentation with overlap for vibration signal.

For the CWRU and PU datasets, we adopt a data augmentation method, which alleviates the difficulty of Few-Shot Learning [39]. The data enhancement technology chooses the overlapping sampling technology. Figure 11 shows the overlap sampling technique.

## 5. Performance Verification

### 5.1. Comparison Settings with Other Methods

The MLKDCE-PBiLSTM algorithm is implemented by the PyTorch library under Python 3.7. Model training and testing of the network are performed on workstations with Windows operating system, Intel Core i7-9750H CPU, and GTX 1660Ti GPU. In the whole process, the network learning rate is 0.001, and the batch size is 32.

We compared the proposed MLKDCE-PBiLSTM with five advanced methods. They are a DCAE network with five-layer convolutional network [15], BiLSTM network [24], LSTM with multiple CNN [23], MSCNN [40], LeNet-5 with a new convolutional neural network proposed by Wen [41]. The six methods adopt the same training strategies in the overall experiments. All datasets are input into the network in 2D form, the MSWT, −1 to 1 normalization, random data segmentation, and augmentation is performed. In the comparative experiment.

The neural network based on multiscale learning proposed by Jiang et al. [40] solved the problem of fault diagnosis of rotating machinery and achieved satisfactory results. Our proposed method has the following differences from the above structure.

1. Multilocation learning: The MLKDCE-PBiLSTM employs skip connections in the branch network to perform multilocation feature learning. The MSCNN neural network employs multiscale coarse-grained operations to down-sample the raw signal, which is probable to lose some features of the input signals.
2. Multikernal operation: In the MSCNN structure, three branches are copy networks, and the extraction of information is insufficient. However, MLKDCE-PBiLSTM uses multiple parallel encoder branches with different convolution kernels and network parameters to extract multiscale fault features.
3. Multifeature fusion: MSCNN does not adopt any feature fusion method, and directly puts the learned features into the final classification layer. The MLKDCE-PBiLSTM uses a multifeature fusion layer to optimize the fusion and optimization of the characteristics learned from multilocation learning and multiscale learning. The network scheme improves the accuracy of the model.
4. Multifeature integration and protection operation: The MLKDCE-PBiLSTM uses a multifeature protection layer to extract long-term dependent fault information in the vibration signal after multifeature integration processing. It is used to maximize the integrity and accuracy of the fault features. However, other comparison networks directly perform dropout or classification operations, which will affect the accuracy or even lose important information.

The fault diagnosis problem studied in this article is essentially a multi-class classification task (PU dataset is 13 categories, CWRU dataset is 10 categories). We use the indicator of accuracy, which is a generally comprehensive indicator metric defined in (25).

(25) $$\mathrm{Accuracy}=\frac{TP+TN}{TP+FN+FP+TN}$$

where *TP* and *TN* refer to the numbers of true positive classes and true negative classes respectively. *FP* and *FN* denote the number of and false positive classes, false negative classes. The indicator ranges from 0 to 1. The larger the value of accuracy, the better the fault diagnosis performance.

### 5.2. Performance Comparison with Other Advanced Methods

Domain adaptation is a significant bearing diagnosis task under variable loads and speeds. It uses the knowledge gained from the training dataset to improve the performance of the network in the test dataset, that is, the study is one of the transfer learning. We cannot obtain the data and distribution of rolling bearing under various working conditions. Therefore, the model trained under the existing load states should accurately diagnose the faults under the new working condition. In this case, the training data and test data should conform to the same characteristic domain and class domain, but the characteristic distribution is inconsistent. In the real world, both the inconsistency of characteristic distribution and the inconsistency of characteristic domain and class domain objectively exist. The above contents are the research content of transfer learning. In this article, two kinds of datasets are used to verify the network domain adaptability.

#### 5.2.1. Comparison Experiment under PU Dataset

We design five experiments, 0.7–0.7 Nm (A), 0.1–0.1 Nm (B), 0.7/0.1–0.1/0.7 Nm (C), 0.1–0.7 Nm (D), and 0.7–0.1 Nm (E) experiments. 0.7–0.1 Nm means that the training dataset is 0.7 Nm load under the speed of 900 rmp, and the test dataset is 0.1 Nm load under 1500 rmp speed, others are similar. This experiment setup can not only verify that the data feature distribution of the training dataset and the test dataset is consistent, but also verify that the feature distribution is inconsistent.

**Figure 12:**
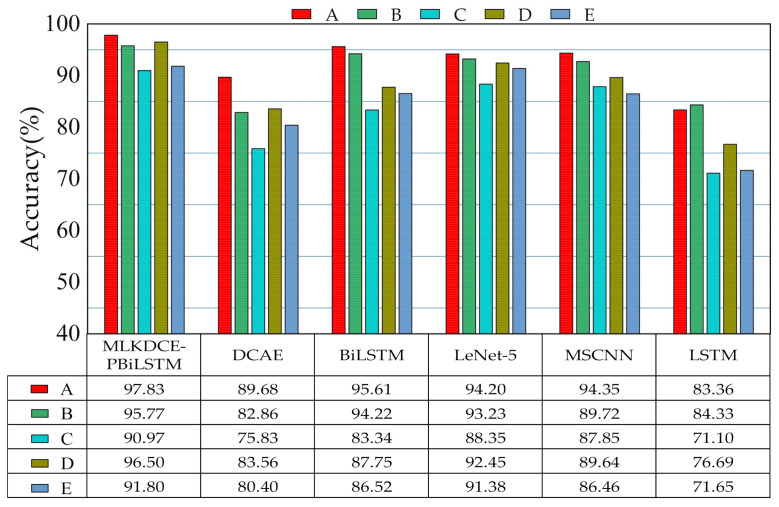
Generalization ability of six comparison methods in the load and speed adaptation of PU dataset.

The testing results repeating three times are shown in Figure 12. Obviously, the MLKDCE-PBiLSTM has obtained the best average diagnosis results among the five tasks of the domain adaptivity under varying loads and speeds. The average accuracy reaches 94.57%. The phenomenon indicates that when the working condition changes, the MLKDCE-PBiLSTM has better domain adaptability of load and speed without using specific domain adaptation methods.

We analyze the accuracy trend of the experimental results from three aspects. (1) The performance of six models in the prediction experiments of group A and B is better than the prediction accuracy of group C, D, and E, respectively. This can be explained by using the consistent distribution of nonlinear data features. (2) The maximum experiment accuracy appears in groups A and B, the minimum experiment accuracy appears in group C, and the accuracy of group D and E is between the above-mentioned load accuracy. This can be explained by the concept of subspace learning. The more the similarity of the subspace, the higher the accuracy of the prediction. In the test of A and B, the principal component features distribution of the two data is consistent, and the correlation is extremely high, so the accuracy of the diagnosis result will be high. In the C group test, the training dataset and the test dataset are a mixed distribution of multiple loads features that the complexity of its subspace is the highest, and the correlation analysis becomes complicated. Therefore, the experiment accuracy compared with the above same characteristic distribution will decrease. For the test experiment of D and E group, from the training dataset to the test dataset is from one feature distribution to another, the accuracy is higher than the above situation. (3) The accuracy of the test experiment D group is always higher than the accuracy of the E group. Basing the vibration knowledge of rotating machinery in dynamics, the smaller the external excitation is, the smaller the rolling bearing vibration response is, and the corresponding vibration characteristics are weaker too. Especially, the load and speed represent the external excitation force. Thus, the discriminant vibration characteristics extracted from a bigger excitation may be weak in a smaller excitation. Contrarily, the discriminant vibration characteristics extracted from a smaller excitation are usually retained in a bigger excitation. Therefore, in the bearing fault diagnosis test, the accuracy of the E group is lower than that of the D group.

The accuracy of the MLKDCE-PBiLSTM in the C group is only 90.97%. Although the accuracy was not as high as under other working cases, the MLKDCE-PBiLSTM result is the best compared with the other five calculation models. The result indicates that our network has a strong generalization ability. Also, MSCNN and LeNeT-5 have better domain adaptability than the other three comparison models. The accuracy shows that multiscale learning can efficiently extract abundant and abstract fault features from the input signals. However, the accuracy of the above two models is 3% and 2% lower than that of the MLKDCE-PBiLSTM. It again indicates that MLFL and MKFL have stronger feature learning ability and feature fusion ability. Besides, an interesting phenomenon can be discovered that BiLSTM also obtains outstanding performance under various load domain adaptations. Thus, we think it is an available neural network with stronger feature extraction ability. In summary, the MLKDCE-PBiLSTM shows the best fault diagnosis performance and generalization capability in the domain adaptation test.

#### 5.2.2. Comparison Experiment under CWRU Dataset

Datasets of F–J contain 10 bearing fault conditions under 1, 2, and 3 hp load. For the datasets of H, all samples of 1 hp and 3 hp are employed as the training set and 2 hp are employed as the test set. For datasets of I and J, the training data is obtained under the load of 1 hp and 3 hp, respectively, and 3 hp and 1 hp are used as the test data.

**Figure 13:**
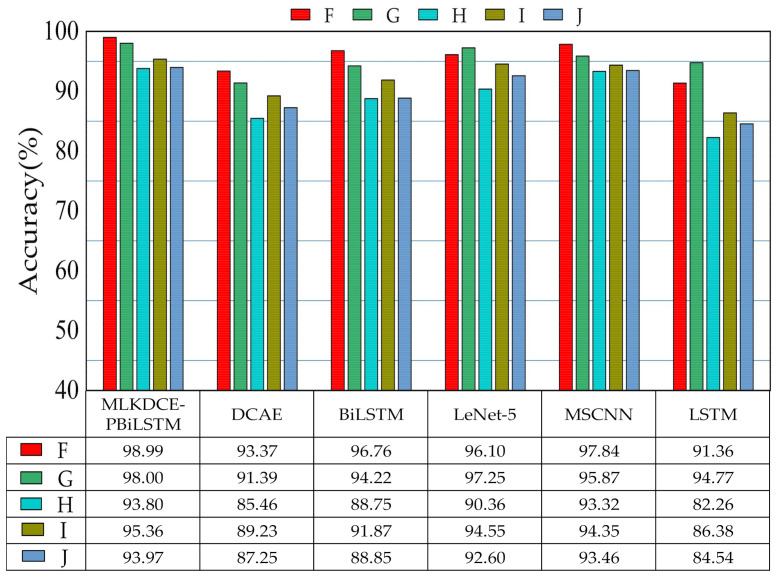
Generalization ability of six comparison methods in the load and speed adaptation of CWRU dataset.

Similar to the above PU tests, the testing results repeating three times are shown in Figure 13. Unsurprisingly, the MLKDCE-PBiLSTM achieves the best average diagnostic results among the five tasks of the domain adaptivity under varying loads and speeds. The average accuracy reaches 96.02%, which indicates that the proposed fault diagnosis system has good domain adaptation and generalization ability. (1) Under the datasets of PU and CWRU, the six models have the same trend of fault diagnosis accuracy under five load cases. (2) However, it is observed that the accuracy of each model has been significantly improved under the CWRU dataset compared with the PU dataset, and its accuracy mostly has reached more than 85%. On the one hand, this situation may be account for the low frequency of CWRU data collection. The upper and lower peaks of the signal are missed during low-frequency sampling, which may weaken the coupling ability of complex signals. On the other hand, compared to the 13 category tasks of the PU dataset, the CWRU dataset is 10 category tasks. These may be the reason for the high accuracy of the model when using the CWRU dataset under variable loads and variable speeds. (3) The experiment accuracy of the MLKDCE-PBiLSTM network is 93.80% under the most complicated experiment H, and in the tests of I and J, the test accuracy reached 95.36% and 93.97%, respectively. Even in the test of F and G, the accuracy reached 98.99% and 98.00%. Outstanding performance can verify that MLKDCE-PBiLSTM possesses the extraction ability of the diverse features.

#### 5.2.3. Computational Burden of the Networks

The testing time of the six models is shown in Table 6. They are all measured under the same software and hardware conditions. The test software is Python 3.7, and the hardware system is Windows operating system, Intel Core i7-9750H CPU, and GTX 1660Ti GPU. The table records the testing time in an epoch. We can observe that the MLKDCE-PBiLSTM consumes more time in all models. It is acceptable for the complex framework with MLFL and MKFL modules.

### 5.3. Verify the Necessity of Each Component of the Model

The performance of a rolling bearing fault diagnosis system has close relations with the quality of network learning outcomes. The core contribution of the MLKDCE-PBiLSTM is to learn and fusion various discriminative fault features with multilocation and multiscale learning; finally, the feature is fed into a multifeature protection module. To accurately evaluate the result of feature learning advantages of each part of the model and the ability to fuse abundant and complementary features, we will explore the impact of different scales features on the classification effect from the following three aspects. The domain adaptation experiments of load and speed are implemented under the data of groups C and H, and the test accuracy of each epoch is shown in the process.

To facilitate the representation of the network structure in the subsequent research, four basic modules of the encoder are set up, including DCNN-M0 (only the basic CNN), MLS-M1 (with skip connection based on the M0), MLS-M2 (only the DCE), and MLS-M3 (with skip connection based on M2). We name the multilocation scale deep convolution encoder as MLDCE.

#### 5.3.1. Necessity of the Multilocation Scale Learning

To accurately evaluate the performance of MLS learning, four network structure, including MLDCE-M0, MLDCE-M1, MLDCE-M2 and MLDCE-M3, are set in this experiment.

**Figure 14:**
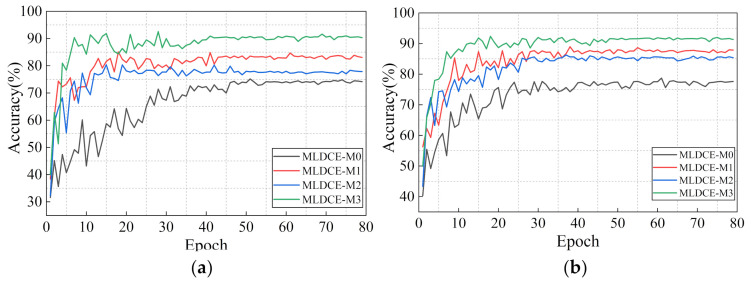
Performance of different MLS modules in each epoch. (**a**) Load and speed experiments in PU dataset; (**b**) Load and speed experiments in CWRU dataset.

The experiment results are shown in Table 7 (average accuracy in the last 10 epochs). Figure 14a,b are the test results of each epoch under variable load and speed conditions of the PU dataset and CWRU dataset, respectively. Obviously, it can be seen from Figure 14a that the diagnostic performance of MLDCE-M2 is higher than that of MLDCE-M0. The result indicates that the deep convolution encoder has stronger feature extraction and compression capabilities. The accuracy of MLDCE-M1 compared to MLDCE -M0 and the accuracy of MLDCE-M3 compared to MLDCE-M2 are improved by nearly 9% and 13%, respectively. The results indicate that even if feature extraction capabilities of the basic network structure with M2 and M0 are weak, MLFL can still extract precise and detailed features (local feature), with invariant robust features (global feature). It effectively improves the network ability of the discriminative fault features extraction. This further demonstrates that the MLKDCE-PBiLSTM with MLFL has significant advantages over traditional encoders. This also proves that the designed MLS module has a better mining ability of detailed features.

According to Figure 14b, compared with the PU experiment, the test result of the CWRU experiment fluctuates greatly, and it takes a longer time to stabilize. Finally, the test accuracy of the MLDCE-M0 module is 77.63%, while the test accuracy of the MLDCE-M3 is 91.32%. From the overall test results, the proposed multiple modules are effective in extracting bearing fault features.

#### 5.3.2. Necessity of the Multikernel Scale Learning

To accurately evaluate the performance of MKS learning, three parallel deep encoder network structures, including MKDCE-B1(n = 1), MKDCE-B2(n = 2), and MKDCE-B3(n = 3), are set in this experiment. It is worth noting that the abovementioned three MKS networks do not include the MLS modules, so we call them multikernel scale deep convolution encoder (MKDCE).

**Figure 15:**
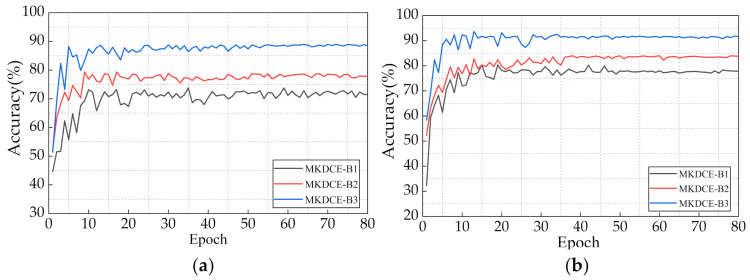
Performance of different MKS modules in each epoch. (**a**) Load and speed experiments in PU dataset; (**b**) Load and speed experiments in CWRU dataset.

The test results are shown in Table 8 (average accuracy in the last 10 epochs). Figure 15a,b are the test results of each epoch under variable loads and speeds conditions of the PU dataset and CWRU dataset, respectively. It is obvious that in Figure 15a, the MKLF module performs stable load adaptation in PU dataset. The module can learn more domain invariant features related to bearing faults from the different kernel sizes of multiple branches. The accuracy of MKDCE-B2 is nearly 6% higher than that of MKDCE-B1, and that of MKDCE-B3 is nearly 10% higher than that of MKDE-B2. Similarly, according to Figure 15b, the accuracy of MKDCE-B2 is nearly 6% higher than that of MKDCE -B1, and that of MKDCE -B3 is nearly 8% higher than that of MKDCE-B2 in the CWRU dataset. It can be seen that the diagnostic accuracy of the network does not increase linearly with the increase of the number of network branches, which indicates that the accuracy of the model can be nonlinearly improved with the use of more parallel MKS branches.

It can be seen that the performance of network mapping is enhancing along with the network width. That is, this advantage will be accumulated in the whole model by more network branches. In practical applications, we can select an appropriate number of MKLF branches for testing according to our own needs and hardware configuration.

#### 5.3.3. Necessity of the Fault Multifeature Fusion

To accurately evaluate the performance of the multifeature fusion, the MLKDCE, MLKDCE-NLF (no multilocation fusion layer in the MLS), the MLKDCE-NBF (no multikernel feature fusion layer in the MKS), and the MLKDCE-NLF-BF (no multilocation and multikernel feature fusion layer) are set in this experiment. The GMSL structure is designed in the four networks.

**Figure 16:**
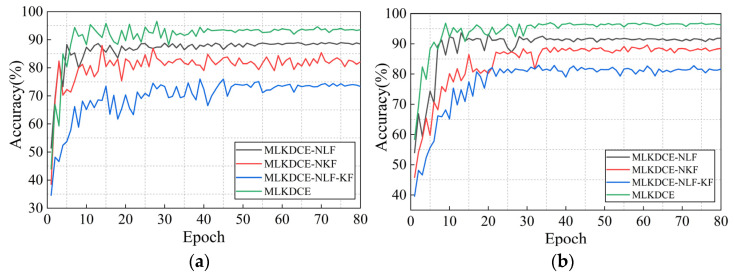
Performance of multifeature fusion modules with respect to epoch. (**a**) Load and speed experiments in PU dataset; (**b**) Load and speed experiments in CWRU dataset.

The test results are shown in Table 9 (average accuracy in the last 10 epochs). Figure 16a,b are the test results under varying load and speed conditions of the PU dataset and CWRU dataset, respectively. In Figure 16a, there are multiple feature distributions of 0.1 Nm and 0.7 Nm in the PU training dataset, so the encoder needs to mining more discriminative features to adapt to the variable load fault diagnosis task. Obviously, although the structure of sample data is complex, the model can better diagnose the bearing fault features. Therefore, the feature fusion layer of the proposed can fuse and optimize the rolling bearing fault features learned from different network locations and different kernel sizes. The MLKDCE-PBiLSTM can extract rich discriminative features from a large amount of bearing data. It indicates that the fusion layer of the composite network has a stronger ability of the robust feature representation strategy in fault pattern classification. Under the variable load and speed of test C in PU, the accuracy of MLKDCE is improved by nearly 13.6% than that of MLKDCE-NLF-KF. Similarly, according to Figure 16b, under the test of group H in CWRU, the accuracy of MLKDCE is improved by nearly 15% compared with MLKDCE-NLF-KF. This fully demonstrates that the MLFL module and MKFL module play a crucial part in the network structure.

#### 5.3.4. Necessity of the Multifeature Protection

To accurately evaluate the performance of multifeature protection, the MLKDCE-PBiLSTM and MLKDCE (no multifeature protection layer before the classification layer) are set in this section.

**Figure 17:**
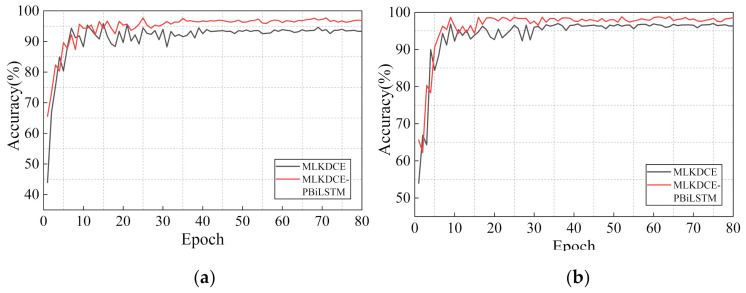
Performance of the multifeature protection module with respect to epoch. (**a**) Load and speed experiments in PU dataset; (**b**) Load and speed experiments in CWRU dataset.

The test results are shown in Table 10 (average accuracy in the last 10 epochs). Figure 17a,b are the test results of each epoch under variable load and speed conditions of the PU dataset and CWRU dataset, respectively. An effective multifeature protection and integration mechanism is necessary for the bearing fault diagnosis system. It can prevent the feature information from being directly fed into the classification layer and losing the integrity of some features. Obviously, the accuracy of MLKDCE-PBiLSTM is nearly 3.3% better than that of MLKDCE in Figure 17a and nearly 2% better than that of MLKDCE in Figure 17b. The accuracy indicates that the multifeature protection layer can deeply extract and integrate unexcavated fault features from the multifeature fusion layer that are more sensitive and dependent signal features. MLKDCE-PBiLSTM performs more stability than MLKDCE under varying operating conditions. It indicates that the PBiLSTM network deals with the abnormal points in the sequence image signal reasonably, and improves the classification effectiveness.

## 6. Conclusions

To extract the multiscale and sensitive feature from the complicated vibration signals, this article proposes a novel MLKDCE-PBiLSTM scheme suitable for the rolling bearing intelligent fault diagnosis under varying conditions of load and speed.

Unlike the traditional multiscale structure, MLKDCE-PBiLSTM combines the skip layer and the last layer of the encoder in each branch, which uses MKS and MLS modules in all GMSL branches. In this way, the multiscale features have stronger invariance and robustness (global features) with precise details (local features). Then, the former network of MLKDCE-PBiLSTM is fed into the feature protection layer for further mining sensitive and complementary features. The multifeature protection layer can deeply mine and protect weak and sensitive fault feature information from the high-purity feature representation of multiple signal components (not directly from the original data). Thus, the MLKDCE-PBiLSTM architecture can effectively diagnose the fault states of the rolling bearings. Compared with the five latest networks with respect to the load and speed adaptability, our method is more accurate and robust. Experimental results prove that multilocation scale module, multikernel scale module, multifeature fusion, and multifeature protection layer can significantly improve the performance of traditional encoders. Therefore, the MLKDCE-PBiLSTM architecture is convinced to be effective applied in the field of intelligent fault diagnosis on the rolling bearings.

In future work, we intend to optimize the network structure for reducing the number of parameters and improve the model stability. In addition, in recent years, deep learning methods have been increasingly applied in the fault diagnosis field. Embedded learning is booming, which is a combination of software and hardware. However, the application of deep learning in embedded systems is less. We want to integrate deep learning into embedded learning for the bearing fault diagnosis in the future. The robustness and effectiveness of the proposed method make it promising and possible for fault diagnosis.

## Figures and Tables

**Table 1:** Description of four working conditions in PU bearing datasets

Setting Name	Rotational Speed (rpm)	Load Torque (nm)
M07_N15_ F10	1500	0.7
M07_N09_ F10	900	0.7
M01_N15_ F10	1500	0.1
M07_N15_F04	1500	0.7

**Table 2:** Details of the used PU bearing datasets.

Name	Fault Location	Fault Description
K001	Healthy	
KA04	Outer ring	Fatigue: pitting
KA15	Outer ring	Plastic deform: indentations
KA22	Outer ring	Fatigue: pitting
KA30	Outer ring	Plastic deform: Indentations
KI18	Inner ring	Fatigue: pitting
KI21	Inner ring	Fatigue: pitting
KI16	Inner ring	Fatigue: pitting
KI04	Inner + outer	Fatigue: pitting; Plastic deform: indentations
KI14	Inner + outer	Fatigue: pitting; Plastic deform: indentations
KB23	Outer + inner	Fatigue: pitting
KB27	Outer + inner	Plastic deform: indentations
KA16	Outer +outer	Fatigue: pitting
KI17	Inner + inner	Fatigue: pitting

**Table 3:** Configuration details of training and testing load for PU datasets.

Index	Loads(nm) of Training/Testing	Speeds	N*_train_*	N*_test_*	Category
A	0.7/0.7	900/900	4800	800	13
B	0.1/0.1	1500/1500	4800	800	13
C	(0.1,0.7)/(0.1,0.7)	(1500,900)/(1500,900)	4800	800	13
D	0.1/0.7	1500/900	4800	800	13
E	0.7/0.1	900/1500	4800	800	13

**Table 4:** CWRU Drive end bearing parameters of SKF62052-RS (diameter size: inches).

Inside	Ball	Outside	Thickness	Pitch
0.9843	0.3126	2.0472	0.5906	1.537

**Table 5:** Configuration details of training and testing load for CWRU datasets.

Index	Loads(hp) of Training/Testing	Speeds(rmp)	N*_train_*	N*_test_*
Normal	/	1796	4800	800
F	1/1	1772	4800	800
G	3/3	1730	4800	800
H	(1,3)/2	(1772,1730)/1750	4800	800
I	1/3	1772/1730	4800	800
J	3/1	1730/1772	4800	800

**Table 6:** Testing time of each epoch in six comparison methods.

	MLKDCE-PBiLSTM	DCAE	BiLSTM	LeNet-5	MSCNN	LSTM
PU	1.8151	1.6007	0.7243	0.7553	0.8365	0.4496
CWRU	2.7327	2.5140	1.1260	1.3548	1.5623	1.1496

**Table 7:** Testing result of different MLS module.

Accuracy (%)	MLDCE-M0	MLDCE-M1	MLDCE-M2	MLDCE-M3
PU Load	74.277	83.198	77.668	90.522
CWRU Load	77.199	87.468	85.303	91.522

**Table 8:** Testing result of different MKS module.

Accuracy (%)	MKDCE-B1	MKDCE-B2	MKDCE-B3
PU Load	72.039	78.009	88.702
CWRU Load	77.668	83.509	91.360

**Table 9:** Testing result of four fault feature fusion cases.

Accuracy (%)	MLKDCE-NLF	MLKDCE-NKF	MLKDCE-NLF-KF	MLKDCE
PU Load	88.702	82.492	79.911	93.522
CWRU Load	91.360	88.114	81.492	96.512

**Table 10:** Testing result of the multifeature protection network.

Accuracy (%)	MLKDCE	MLKDCE-PBiLSTM
PU Load	93.522	96.795
CWRU Load	96.522	97.946

## Data Availability

The data presented in this study are openly available in Case Western Reserve University and Universität Paderborn databases.
